# Benzyl­sulfamide

**DOI:** 10.1107/S1600536811019490

**Published:** 2011-05-28

**Authors:** Thomas Gelbrich, Mairi F. Haddow, Ulrich J. Griesser

**Affiliations:** a Institute of Pharmacy, University of Innsbruck, Innrain 52, 6020 Innsbruck, Austria

## Abstract

The crystal of the title compound [systematic name: 4-(benzyl­amino)­benzene­sulfonamide], C_13_H_14_N_2_O_2_S, displays a hydrogen-bonded framework structure. Mol­ecules are doubly N—H⋯O hydrogen bonded to one another *via* their NH_2_ groups and sulfonyl O atoms. These inter­actions generate a hydrogen-bonded ladder structure parallel to the *a* axis, which contains fused *R*_2_^2^(8) rings. The NH group serves as the hydrogen-bond donor for a second set of inter­molecular N—H⋯O=S inter­actions.

## Related literature

For the pharmacology and synthesis of the title compound, see Goissedet *et al.* (1936 ▶); Goissedet & Despois (1938 ▶); Mellon *et al.* (1938 ▶); Long & Bliss (1939 ▶). For related structures, see: Hursthouse *et al.* (1998 ▶, 1999*a* ▶,*b* ▶); Gelbrich *et al.* (2008 ▶); Davis *et al.* (1996 ▶); Costanzo *et al.* (1999 ▶); Kubicki & Codding (2001 ▶);Yathirajan *et al.* (2005 ▶); Denehy *et al.* (2006 ▶); Toumieux *et al.* (2006 ▶). For graph-set analysis, see: Bernstein *et al.* (1995 ▶).
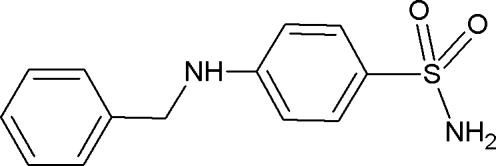


## Experimental

### 

#### Crystal data

C_13_H_14_N_2_O_2_S
*M* *_r_* = 262.32
Orthorhombic, 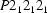

*a* = 7.8426 (1) Å
*b* = 10.5549 (11) Å
*c* = 14.6694 (3) Å
*V* = 1214.30 (13) Å^3^
*Z* = 4
Mo *K*α radiation
μ = 0.26 mm^−1^
*T* = 120 K
0.20 × 0.20 × 0.15 mm

#### Data collection

Bruker–Nonius Roper CCD camera on κ-goniostat diffractometer
Absorption correction: multi-scan (*SADABS*; Sheldrick, 2007 ▶) *T*_min_ = 0.950, *T*_max_ = 0.962
11460 measured reflections
2364 independent reflections
2312 reflections with *I* > 2σ(*I*)
*R*_int_ = 0.027

#### Refinement

*R*[*F* ^2^ > 2σ(*F* ^2^)] = 0.023
*wR*(*F* ^2^) = 0.061
*S* = 1.06
2364 reflections
176 parameters
3 restraints
H atoms treated by a mixture of independent and constrained refinement
Δρ_max_ = 0.19 e Å^−3^
Δρ_min_ = −0.30 e Å^−3^
Absolute structure: Flack (1983 ▶), 972 Friedel pairs
Flack parameter: −0.01 (5)

### 

Data collection: *COLLECT* (Hooft, 1998 ▶); cell refinement: *DENZO* (Otwinowski & Minor, 1997 ▶) and *COLLECT*; data reduction: *DENZO* and *COLLECT*; program(s) used to solve structure: *SHELXS97* (Sheldrick, 2008 ▶); program(s) used to refine structure: *SHELXL97* (Sheldrick, 2008 ▶); molecular graphics: *XP* in *SHELXTL* (Sheldrick, 2008 ▶) and *Mercury* (Bruno *et al.*, 2002 ▶); software used to prepare material for publication: *publCIF* (Westrip, 2010 ▶).

## Supplementary Material

Crystal structure: contains datablocks I, global. DOI: 10.1107/S1600536811019490/ez2246sup1.cif

Structure factors: contains datablocks I. DOI: 10.1107/S1600536811019490/ez2246Isup2.hkl

Supplementary material file. DOI: 10.1107/S1600536811019490/ez2246Isup3.cml

Additional supplementary materials:  crystallographic information; 3D view; checkCIF report

## Figures and Tables

**Table 1:** Hydrogen-bond geometry (Å, °)

*D*—H⋯*A*	*D*—H	H⋯*A*	*D*⋯*A*	*D*—H⋯*A*
N2—H3*N*⋯O2^i^	0.87 (2)	2.20 (2)	3.0264 (16)	160 (2)
N1—H2*N*⋯O1^ii^	0.89 (2)	2.09 (2)	2.9613 (16)	168 (2)
N1—H1*N*⋯O2^iii^	0.86 (1)	2.18 (1)	3.0281 (16)	172 (2)

Symmetry codes: (i) 
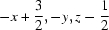
; (ii) 
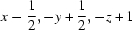
; (iii) 
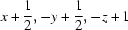
.

**Fig. 1.**
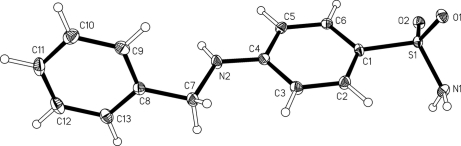
The molecular structure with displacement ellipsoids drawn at the 50% probability level. Hydrogen atoms are shown as spheres of arbitrary size.

**Fig. 2.**
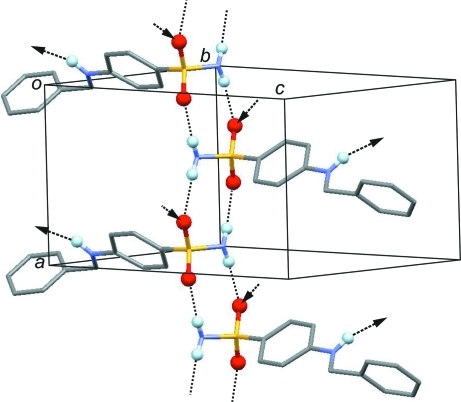
Ladder structure parallel to [100] formed by H-bonds involving the NH2 group. The interactions between the NH group and O2 are indicated by arrows. O and H atoms directly engaged in N–H···O bonds are drawn as balls.

**Fig. 3.**
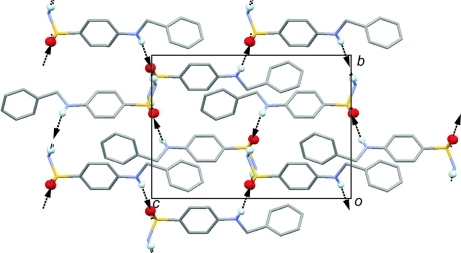
H-bonded framework structure viewed parallel to the a-axis, with H-bonds indicated by arrows.

## supplementary crystallographic information

### Comment

The title compound (synonyms: proseptazine, septazine, benzylsulfanilamide, chemodyn; CAS No. 104–22–3), first marketed in 1936, was one of the early antibacterial agents of the sulfonamide class (Goissedet *et al.*, 1936; Goissedet & Despois, 1938; Mellon *et al.*, 1938; Long & Bliss, 1939). The C–N–(C_6_H_4_)–S fragment of the molecule (see Fig. 1) is essentially planar, and the molecular geometry is characterized by the torsion angles N1–S1–C1–C2 and N2–C7–C8–C9 of 43.6 (1)° and -65.9 (2)°, respectively.

The crystal structure contains three independent intermolecular N—H···O=S bonds which lead to the formation of an H-bonded framework. Each molecule is doubly H-bonded, *via* its NH_2_ and sulfonyl groups, to two neighbouring molecules. These interactions generate an N—H···O=S-bonded ladder structure parallel to [100], which consists of fused *R*^2^_2_(8) rings (Bernstein *et al.*, 1995) and displays a 2_1_ symmetry. This situation is illustrated in Fig. 2. The same one-dimensional structure has been found previously in only a few other compounds of the sulfonamide class, see Davis *et al.* (1996); Costanzo *et al.* (1999); Kubicki & Codding (2001);Yathirajan *et al.* (2005); Denehy *et al.* (2006); Toumieux *et al.* (2006).

The sulfonyl oxygen atom O2 accepts an additional H-bond from the NH group of a neighbouring molecule. This interaction links molecules which are related to one another by a 2_1_ operation parallel to the *c*-axis (see Fig. 3).

### Refinement

All H atoms were identified in a difference map. Secondary CH_2_ (C—H = 0.99 Å) and aromatic carbon atoms (C—H = 0.95 Å) were positioned geometrically and refined with *U*_iso_ = 1.2 *U_eq_*(C). Hydrogen atoms attached to N and O were refined with restrained distances [N—H = 0.88 (2) Å]; and their *U*_iso_ parameters were refined freely.

### Crystal data

**Table:**

C_13_H_14_N_2_O_2_S	*F*(000) = 552
*M**_r_* = 262.32	*D*_x_ = 1.435 Mg m^−^^3^
Orthorhombic, *P*2_1_2_1_2_1_	Mo *K*α radiation, λ = 0.71073 Å
Hall symbol: P 2ac 2ab	Cell parameters from 6120 reflections
*a* = 7.8426 (1) Å	θ = 2.9–27.5°
*b* = 10.5549 (11) Å	µ = 0.26 mm^−^^1^
*c* = 14.6694 (3) Å	*T* = 120 K
*V* = 1214.30 (13) Å^3^	Block, colourless
*Z* = 4	0.20 × 0.20 × 0.15 mm

### Data collection

**Table:**

Bruker–Nonius Roper CCD camera on κ-goniostat diffractometer	2364 independent reflections
Radiation source: Bruker-Nonius FR591 rotating anode	2312 reflections with *I* > 2σ(*I*)
graphite	*R*_int_ = 0.027
Detector resolution: 9.091 pixels mm^-1^	θ_max_ = 26.0°, θ_min_ = 3.2°
φ and ω scans	*h* = −9→9
Absorption correction: multi-scan (*SADABS*; Sheldrick, 2007)	*k* = −12→13
*T*_min_ = 0.950, *T*_max_ = 0.962	*l* = −18→18
11460 measured reflections	

### Refinement

**Table:**

Refinement on *F*^2^	Hydrogen site location: inferred from neighbouring sites
Least-squares matrix: full	H atoms treated by a mixture of independent and constrained refinement
*R*[*F*^2^ > 2σ(*F*^2^)] = 0.023	*w* = 1/[σ^2^(*F*_o_^2^) + (0.032*P*)^2^ + 0.3394*P*] where *P* = (*F*_o_^2^ + 2*F*_c_^2^)/3
*wR*(*F*^2^) = 0.061	(Δ/σ)_max_ = 0.001
*S* = 1.06	Δρ_max_ = 0.19 e Å^−^^3^
2364 reflections	Δρ_min_ = −0.30 e Å^−^^3^
176 parameters	Extinction correction: *SHELXS97* (Sheldrick, 2008), Fc^*^=kFc[1+0.001xFc^2^λ^3^/sin(2θ)]^-1/4^
3 restraints	Extinction coefficient: 0.021 (4)
Primary atom site location: structure-invariant direct methods	Absolute structure: Flack (1983), 972 Friedel pairs
Secondary atom site location: difference Fourier map	Flack parameter: −0.01 (5)

### Special details

**Table:**

Geometry. All e.s.d.'s (except the e.s.d. in the dihedral angle between two l.s. planes) are estimated using the full covariance matrix. The cell e.s.d.'s are taken into account individually in the estimation of e.s.d.'s in distances, angles and torsion angles; correlations between e.s.d.'s in cell parameters are only used when they are defined by crystal symmetry. An approximate (isotropic) treatment of cell e.s.d.'s is used for estimating e.s.d.'s involving l.s. planes.
Refinement. Refinement of *F*^2^ against ALL reflections. The weighted *R*-factor *wR* and goodness of fit *S* are based on *F*^2^, conventional *R*-factors *R* are based on *F*, with *F* set to zero for negative *F*^2^. The threshold expression of *F*^2^ > σ(*F*^2^) is used only for calculating *R*-factors(gt) *etc*. and is not relevant to the choice of reflections for refinement. *R*-factors based on *F*^2^ are statistically about twice as large as those based on *F*, and *R*- factors based on ALL data will be even larger.

### Fractional atomic coordinates and isotropic or equivalent isotropic displacement parameters (Å^2^)

**Table:**

	*x*	*y*	*z*	*U*_iso_*/*U*_eq_	
S1	0.88925 (4)	0.14891 (3)	0.47921 (2)	0.00933 (11)	
O1	1.04460 (12)	0.09326 (9)	0.51381 (7)	0.0132 (2)	
O2	0.73029 (12)	0.08853 (9)	0.50432 (7)	0.0127 (2)	
N1	0.88280 (16)	0.29082 (11)	0.51938 (8)	0.0124 (3)	
H2N	0.789 (2)	0.3327 (18)	0.5037 (13)	0.027 (5)*	
H1N	0.9760 (19)	0.3323 (16)	0.5125 (12)	0.016 (4)*	
N2	0.94618 (16)	0.14533 (12)	0.07743 (8)	0.0141 (3)	
H3N	0.879 (2)	0.0925 (18)	0.0498 (13)	0.032 (5)*	
C1	0.89793 (18)	0.15385 (13)	0.36038 (9)	0.0103 (3)	
C2	1.00520 (19)	0.23999 (13)	0.31668 (10)	0.0131 (3)	
H2	1.0688	0.2990	0.3518	0.016*	
C3	1.01965 (19)	0.24017 (13)	0.22311 (10)	0.0135 (3)	
H3	1.0908	0.3008	0.1940	0.016*	
C4	0.92945 (17)	0.15099 (14)	0.17015 (9)	0.0110 (3)	
C5	0.82057 (18)	0.06548 (14)	0.21519 (10)	0.0115 (3)	
H5	0.7574	0.0057	0.1805	0.014*	
C6	0.80389 (18)	0.06679 (13)	0.30896 (9)	0.0107 (3)	
H6	0.7291	0.0090	0.3383	0.013*	
C9	1.15434 (18)	0.09176 (14)	−0.09378 (10)	0.0145 (3)	
H9	1.2109	0.0486	−0.0456	0.017*	
C7	1.03437 (19)	0.24066 (13)	0.02344 (10)	0.0138 (3)	
H7A	1.1487	0.2564	0.0497	0.017*	
H7B	0.9696	0.3211	0.0251	0.017*	
C8	1.05223 (18)	0.19639 (14)	−0.07416 (10)	0.0119 (3)	
C10	1.1744 (2)	0.04982 (15)	−0.18294 (11)	0.0182 (3)	
H10	1.2445	−0.0214	−0.1957	0.022*	
C11	1.0911 (2)	0.11273 (15)	−0.25348 (10)	0.0187 (3)	
H11	1.1038	0.0840	−0.3145	0.022*	
C12	0.9903 (2)	0.21671 (15)	−0.23489 (11)	0.0184 (3)	
H12	0.9340	0.2596	−0.2832	0.022*	
C13	0.97056 (19)	0.25909 (14)	−0.14530 (10)	0.0150 (3)	
H13	0.9013	0.3309	−0.1329	0.018*	

### Atomic displacement parameters (Å^2^)

**Table:**

	*U*^11^	*U*^22^	*U*^33^	*U*^12^	*U*^13^	*U*^23^
S1	0.00952 (18)	0.01096 (17)	0.00751 (17)	0.00004 (13)	0.00065 (13)	0.00008 (13)
O1	0.0124 (5)	0.0159 (5)	0.0115 (5)	0.0029 (4)	−0.0014 (4)	0.0013 (4)
O2	0.0118 (5)	0.0143 (5)	0.0120 (5)	−0.0022 (4)	0.0026 (4)	0.0015 (4)
N1	0.0110 (6)	0.0127 (6)	0.0136 (6)	−0.0003 (5)	0.0009 (6)	−0.0031 (5)
N2	0.0181 (6)	0.0152 (6)	0.0090 (6)	−0.0072 (5)	0.0009 (5)	0.0005 (5)
C1	0.0105 (6)	0.0132 (7)	0.0073 (6)	0.0018 (6)	0.0007 (5)	0.0008 (5)
C2	0.0137 (7)	0.0134 (7)	0.0122 (7)	−0.0033 (6)	−0.0011 (6)	−0.0012 (6)
C3	0.0145 (7)	0.0136 (7)	0.0125 (7)	−0.0049 (6)	0.0011 (6)	0.0017 (6)
C4	0.0117 (6)	0.0116 (6)	0.0099 (6)	0.0011 (6)	0.0000 (5)	0.0004 (6)
C5	0.0109 (7)	0.0114 (6)	0.0123 (6)	−0.0016 (5)	−0.0006 (5)	−0.0019 (6)
C6	0.0106 (7)	0.0099 (6)	0.0117 (6)	−0.0007 (5)	0.0008 (5)	0.0006 (6)
C9	0.0132 (7)	0.0150 (7)	0.0154 (7)	−0.0002 (6)	−0.0015 (5)	0.0027 (6)
C7	0.0167 (7)	0.0139 (7)	0.0109 (7)	−0.0039 (6)	0.0016 (6)	0.0016 (6)
C8	0.0117 (7)	0.0128 (6)	0.0112 (7)	−0.0047 (5)	0.0002 (5)	0.0006 (6)
C10	0.0176 (8)	0.0158 (7)	0.0212 (8)	−0.0008 (6)	0.0041 (6)	−0.0029 (6)
C11	0.0220 (8)	0.0236 (8)	0.0105 (7)	−0.0104 (6)	0.0039 (6)	−0.0030 (6)
C12	0.0187 (8)	0.0236 (8)	0.0129 (8)	−0.0059 (6)	−0.0035 (6)	0.0058 (6)
C13	0.0128 (7)	0.0175 (8)	0.0146 (7)	0.0006 (6)	0.0002 (6)	0.0029 (6)

### Geometric parameters (Å, °)

**Table:**

S1—O1	1.4447 (10)	C5—H5	0.9500
S1—O2	1.4477 (10)	C6—H6	0.9500
S1—N1	1.6104 (12)	C9—C10	1.390 (2)
S1—C1	1.7452 (13)	C9—C8	1.394 (2)
N1—H2N	0.889 (15)	C9—H9	0.9500
N1—H1N	0.858 (14)	C7—C8	1.5126 (19)
N2—C4	1.3677 (17)	C7—H7A	0.9900
N2—C7	1.4553 (17)	C7—H7B	0.9900
N2—H3N	0.867 (15)	C8—C13	1.392 (2)
C1—C2	1.3948 (19)	C10—C11	1.392 (2)
C1—C6	1.399 (2)	C10—H10	0.9500
C2—C3	1.377 (2)	C11—C12	1.380 (2)
C2—H2	0.9500	C11—H11	0.9500
C3—C4	1.411 (2)	C12—C13	1.397 (2)
C3—H3	0.9500	C12—H12	0.9500
C4—C5	1.407 (2)	C13—H13	0.9500
C5—C6	1.3817 (19)		
			
O1—S1—O2	117.25 (6)	C5—C6—C1	119.57 (13)
O1—S1—N1	106.02 (6)	C5—C6—H6	120.2
O2—S1—N1	106.81 (6)	C1—C6—H6	120.2
O1—S1—C1	109.28 (6)	C10—C9—C8	120.77 (14)
O2—S1—C1	107.51 (6)	C10—C9—H9	119.6
N1—S1—C1	109.81 (6)	C8—C9—H9	119.6
S1—N1—H2N	113.2 (13)	N2—C7—C8	110.22 (11)
S1—N1—H1N	113.9 (12)	N2—C7—H7A	109.6
H2N—N1—H1N	114.9 (17)	C8—C7—H7A	109.6
C4—N2—C7	123.82 (12)	N2—C7—H7B	109.6
C4—N2—H3N	115.7 (14)	C8—C7—H7B	109.6
C7—N2—H3N	118.6 (14)	H7A—C7—H7B	108.1
C2—C1—C6	119.88 (13)	C13—C8—C9	119.09 (14)
C2—C1—S1	120.14 (11)	C13—C8—C7	121.35 (13)
C6—C1—S1	119.89 (10)	C9—C8—C7	119.56 (13)
C3—C2—C1	120.57 (13)	C9—C10—C11	119.64 (14)
C3—C2—H2	119.7	C9—C10—H10	120.2
C1—C2—H2	119.7	C11—C10—H10	120.2
C2—C3—C4	120.44 (13)	C12—C11—C10	120.08 (14)
C2—C3—H3	119.8	C12—C11—H11	120.0
C4—C3—H3	119.8	C10—C11—H11	120.0
N2—C4—C5	119.81 (13)	C11—C12—C13	120.27 (15)
N2—C4—C3	121.92 (13)	C11—C12—H12	119.9
C5—C4—C3	118.27 (13)	C13—C12—H12	119.9
C6—C5—C4	121.22 (13)	C8—C13—C12	120.15 (14)
C6—C5—H5	119.4	C8—C13—H13	119.9
C4—C5—H5	119.4	C12—C13—H13	119.9
			
N1—S1—C1—C2	43.59 (14)	N2—C7—C8—C9	−65.94 (17)

### Hydrogen-bond geometry (Å, °)

**Table:**

*D*—H···*A*	*D*—H	H···*A*	*D*···*A*	*D*—H···*A*
N2—H3N···O2^i^	0.87 (2)	2.20 (2)	3.0264 (16)	160.(2)
N1—H2N···O1^ii^	0.89 (2)	2.09 (2)	2.9613 (16)	168.(2)
N1—H1N···O2^iii^	0.86 (1)	2.18 (1)	3.0281 (16)	172.(2)

Symmetry codes: (i) −*x*+3/2, −*y*, *z*−1/2; (ii) *x*−1/2, −*y*+1/2, −*z*+1; (iii) *x*+1/2, −*y*+1/2, −*z*+1.
